# A Review and Comparison of Immune-Checkpoint Inhibitors in the Treatment of Metastatic Uveal Melanoma

**DOI:** 10.3390/jcm14030885

**Published:** 2025-01-29

**Authors:** Benyamin Alam, Amir Reza Akbari, Ahmed Ageed, Ryan Duffy

**Affiliations:** 1 Queen Elizabeth Hospital, Birmingham B15 2WB, UK; 2 King’s Mill Hospital, Sutton-in-Ashfield NG17 4JL, UK; 3 Leicester, Royal Infirmary, Leicester LE1 5WW, UK

**Keywords:** metastatic uveal melanoma, chemotherapy, immune checkpoint inhibitors, objective response, disease control, median progression-free survival, adverse events

## Abstract

**Introduction:** Metastatic uveal melanoma (mUM) is a rare and aggressive malignancy characterised by poor responsiveness to conventional chemotherapies, posing significant treatment challenges. Immune checkpoint inhibitor (ICI) therapies, including monotherapies with Ipilimumab, pembrolizumab, and nivolumab, as well as dual ICI therapy, have emerged as potential treatments. Whilst current research favours dual therapy over single therapy, comprehensive individualised comparisons of the efficacy and safety profiles of these therapies remain limited. This meta-analysis aims to evaluate the clinical outcomes of single ICI therapies individually and compare against combination therapy to guide optimal treatment strategies for mUM. **Methods:** A systematic literature review was conducted to identify studies reporting objective response rates (ORR), disease control rates (DCR), median progression-free survival (MPFS), and adverse event rates (AER) for Ipilimumab, pembrolizumab, nivolumab, and dual ICI therapy. Data were aggregated using forest plots and analysed to compare the efficacy and safety of each regimen. **Results:** Dual ICI therapy demonstrated the highest ORR and DCR but showed no statistically significant advantage over monotherapies. Dual therapy also had a lower MPFS than both pembrolizumab and nivolumab monotherapies. Furthermore, dual therapy was associated with a much greater AER compared to any single therapy, including pembrolizumab and nivolumab. **Conclusions:** While dual ICI therapy offers improved ORR and DCR on aggregate analyses, monotherapies like pembrolizumab provide comparable outcomes in specific metrics, particularly MPFS, with significantly reduced toxicity. These findings underscore the need for personalised ICI regimens tailored to individual patient profiles rather than defaulting to dual therapy. Further research is essential to refine treatment guidelines and optimise outcomes for mUM patients.

## 1. Introduction

### Background

Metastatic uveal melanoma (mUM) is a rare but highly aggressive malignancy originating from melanocytes of the uveal tract [1]. While primary uveal melanoma can often be managed with localised treatments like radiation or surgery, metastatic progression—most commonly to the liver—is associated with poor prognosis and limited therapeutic options [2,3]. The distinct molecular and immunological features of mUM, such as its low mutational burden and immune-evasive tumour microenvironment, underscore the need for tailored therapeutic strategies [4].

Immune checkpoint inhibitor (ICI) therapies have revolutionised cancer treatment, particularly for metastatic cutaneous melanoma. These therapies target immune inhibitory pathways, such as cytotoxic T-lymphocyte-associated antigen 4 (CTLA-4) and programmed cell death protein 1 (PD-1), to enhance anti-tumour immune responses [5,6]. The most common immune checkpoint inhibitors currently in use are Ipilimumab, pembrolizumab and nivolumab [6]. However, the unique biology of mUM, including its low neoantigen expression and immune-privileged environment, contributes to the modest response rates observed with ICI therapies in this disease [7].

Monotherapy with anti-PD-1 agents like nivolumab and pembrolizumab has shown limited efficacy in mUM, with objective response rates (ORRs) ranging from 5% to 15% [8,9]. Similarly, anti-CTLA-4 monotherapy with Ipilimumab has shown even lower response rates [9]. Dual ICI therapy, combining anti-CTLA-4 and anti-PD-1 agents, has demonstrated improved efficacy in preliminary studies, but this comes with increased toxicity, raising questions about the optimal balance between efficacy and safety [10,11].

Tebentafusp, a bispecific T-cell engager, has emerged as a novel and highly effective treatment for HLA-A0201-positive mUM patients, demonstrating superior survival outcomes compared to ICI therapies [12]. However, this treatment is limited to patients with the HLA-A0201 genotype, leaving a significant proportion of patients reliant on ICI therapies [13].

The application of ICI therapies in mUM is hindered by several unresolved questions. Of the limited research conducted, existing meta-analyses have identified improved outcomes with dual ICI therapy when compared to single ICI therapies when combined [10]. However, no current research has separated each single ICI therapy and compared these against each other and against dual therapy. This could answer key questions such as which ICI is superior amongst others when used in isolation, and also whether any particular single therapy can compete or even outperform dual therapy.

This meta-analysis aims to address these gaps by systematically evaluating the efficacy and safety of ICI therapies in mUM. By pooling data across small, underpowered studies, it provides statistically robust insights into the comparative performance of ICI regimens. The findings also address the unmet need for head-to-head comparisons of single-agent therapies, which is crucial given the toxicity associated with dual therapy. We aim to compare each single therapy and dual therapy across the following outcomes: objective response rate (ORR), disease control rate (DCR), median progression-free survival (MPFS) and adverse event rate (AER).

## 2. Materials and Methods

### 2.1. Literature Search

Data collection started with a literature search performed on the following online libraries: Pubmed, Embase, OVID and the Cochrane Library. The search used was devised by stratifying MeSH terms into three distinct categories, the first being population. This group included terms to ensure the participants of the studies adequately met the target population for this meta-analysis. The second group included terms regarding the intervention, so the yielded studies investigated immune checkpoint inhibitors. Finally, the third group was composed of terms describing the outcomes of interest in this analysis, including objective response rate, disease control rate, median progression-free survival and adverse events. Terms within each category were combined using the Boolean ‘OR’ function, and the groups were then combined using the Boolean ‘AND’ function. The MeSH search terms and categories are listed in the Appendix A as Table A1.

Duplicates identified between the different libraries were eliminated. The titles and abstracts of the remaining articles were screened and unsuitable studies were removed. Remaining articles were read in full to assess whether they met the inclusion and exclusion criteria. All studies that passed the above steps were included for data collection

### 2.2. Inclusion and Exclusion Criteria

Exclusion and inclusion criteria were modelled off the PICO structure. Population—Diagnosis of metastatic uveal melanoma; Intervention—ICI therapy; Comparators—N/A; Outcome—ORR, DCR, MPFS, AER; Study design—retrospective, prospective or clinical trial.

The following inclusion criteria were devised:

(1) All participants must have a diagnosis of metastatic uveal melanoma;
(2) All studies must be prospective, retrospective or clinical trials;
(3) The Independent variable must be therapy administered;
(4) All participants within one arm must receive the same chemotherapy regime;
(5) Intention to treat protocol was used;
(6) The study must be available in English.

Below are the exclusion criteria:

(1) Participants within one cohort received treatment with different ICIs;
(2) Duplicate of another study;
(3) Conference abstracts or reviews;
(4) Participants receiving adjuvant/neo-adjuvant chemotherapy or combination therapy with non-ICI regimes;
(5) If the study focused on non-uveal ocular melanomas (e.g., iridial melanomas).

### 2.3. Data Extraction

All articles collected were first evaluated for demographic information such as number of participants, mean age, and gender, followed by treatment regime and follow up period. Following this, data for the primary outcome—objective response rate—and secondary outcomes—disease control rate, median progression-free survival and adverse events. These were collected alongside standard error or confidence intervals where applicable. For each outcome, studies were grouped by ICI regime to compare their efficacies against each other.

### 2.4. Statistical Analysis

#### 2.4.1. Binary Data: ORR, DCR, AE

For outcomes assessing the following binary data: ORR, DCR and AER, the outcome data along with relevant demographic data were entered into ReviewManager5© as per Cochrane guidance [14]. Standard error was estimated using Wilson score interval [15]. A Forest Plot was created for each outcome within each ICI cohort to assess outcome rate with an inverse variance statistical method and fixed effects analysis model. The overall effect values generated from the forest plot for each ICI were compared against each other for these different outcomes to see which treatment resulted in the greatest change.

#### 2.4.2. Non-Binary Data: Median Progression-Free Survival

Non-binary data needed to be handled slightly differently compared to the binary outcomes, as the same statistical analysis would not be applicable. The median survival time and 95% CI were logarithmically transformed. A normal distribution was assumed for the standard error of survival data using the formula proposed by Zang et al. [16]. When this same doctrine could not be applied, the CI was classed as the time during which the upper and lower CI survival proportions worked out to 50% survival. I2 statistics were used to interpret the heterogeneity in a standard manner [17].

### 2.5. Bias Testing

Studies were screened to ensure that none were significantly biased. A quality assessment was conducted in accordance with the Clarity Group of McMasters university guidance [18]. Initially, each study was independently scored by two others (B.A, A.R.A) independently across 6 different domains: selection bias, information bias, attrition bias, sampling bias, detection bias and other sources of bias. Each domain was scored discretely, as either low risk of bias, unclear risk of bias, or high risk of bias. In the event of disagreement, a third assessment was conducted by another author independently (A.A).

## 3. Results

### 3.1. Literature Search

**Figure 1:**
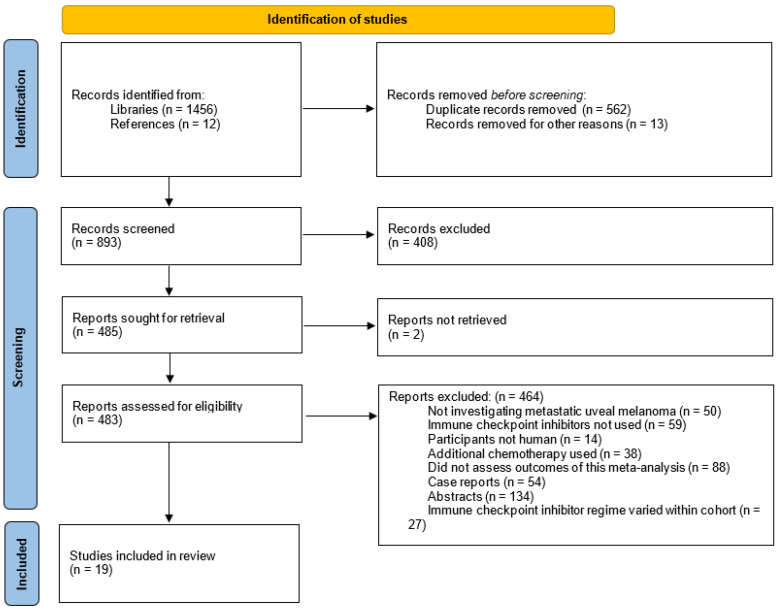
PRISMA chart.

The initial search across all libraries identified a total of 1468 different studies (1456 from libraries and 12 from additional searches of included references). Approximately one-third of these were identified as duplicates with an additional 13 excluded for other reasons, such as not being available in English. This left 893 results to be screened by title and abstract. A total 408 of these were not relevant to this meta-analysis and were excluded; retrieval was sought for the remaining 485 studies, although 2 could not be retrieved. Each of these were then screened to see whether inclusion and exclusion criteria were met. A total of 464 did not meet these criteria and were subsequently discarded. This left a total of 19 studies to be included within this meta-analysis. This process is outlined in greater detail within the PRISMA chart displayed in Figure 1.

This search identified a total of 19 studies that fit the inclusion criteria, resulting in a total of 683 patients analysed. Of these, 239 received Ipilimumab; 163 received pembrolizumab; 62 nivolumab; and 219 belonged to the dual therapy cohort. All of the aforementioned studies and their associated demographic data and outcomes are displayed in Table 1.

### 3.2. Assessment of Bias

**Figure 2:**
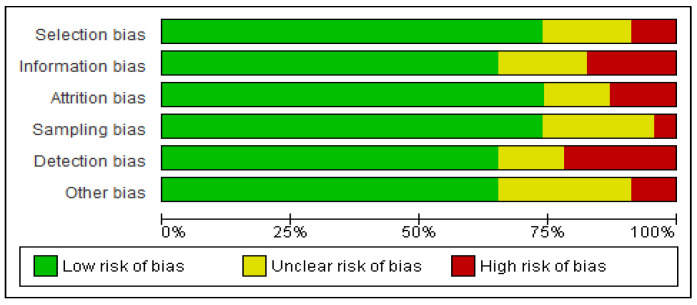
Summary of bias assessment.

After a thorough assessment of bias, the sum of all studies was found to be at low risk of bias. The yielded articles performed similarly across all domains; they scored best in sampling bias, with 75% of all studies identified as low risk of bias. The domain with the lowest score was detection bias, with 65% having a low risk of bias and 20% a high risk of bias; this was as often due to the rationale for criteria for identifying adverse events being ambiguous or follow up periods being variable. A graphic representation of the bias assessment can be seen in Figure 2.

### 3.3. Meta Analysis

#### 3.3.1. Objective Response Rate

**Figure 3:**
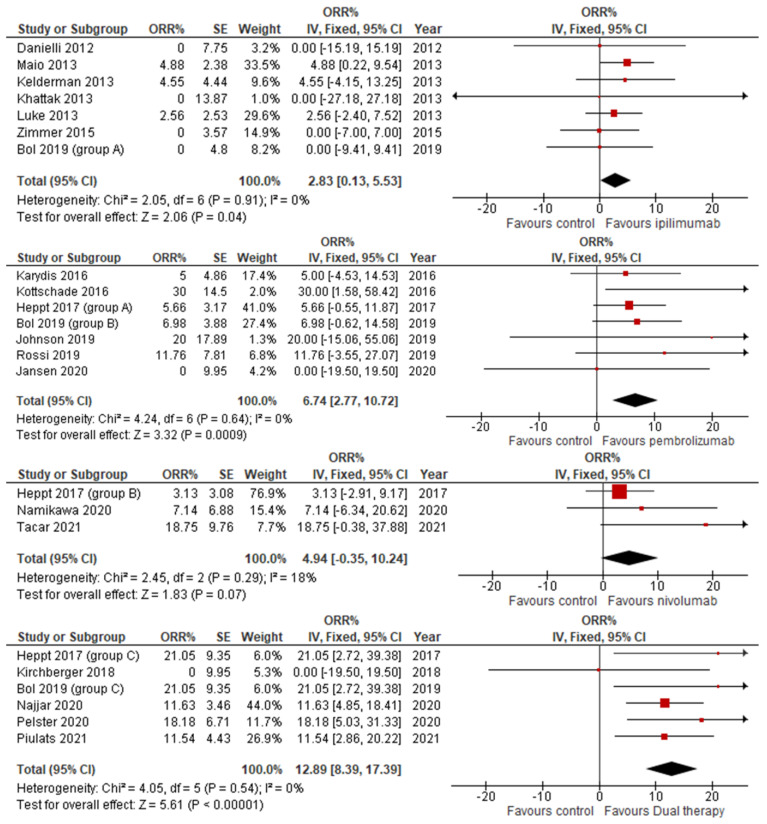
Objective response rate stratified by ICI therapy.

Our results show a varying degree of success in ORR across the different ICIs. All ICIs showed a statistically significant improvement in ORR, with the exception of nivolumab, which crossed the 0 line and therefore could not provably exhibit an improvement in ORR. Of the remaining ICIs, dual therapy had the greatest results, with a combined ORR of 12.89 (CI 8.39–17.39) followed by pembrolizumab at 6.74 (CI 2.77–10.72) then finally Ipilimumab at 2.83 (CI 0.13–5.53). This can be seen in Figure 3.

#### 3.3.2. Disease Control Rate

**Figure 4:**
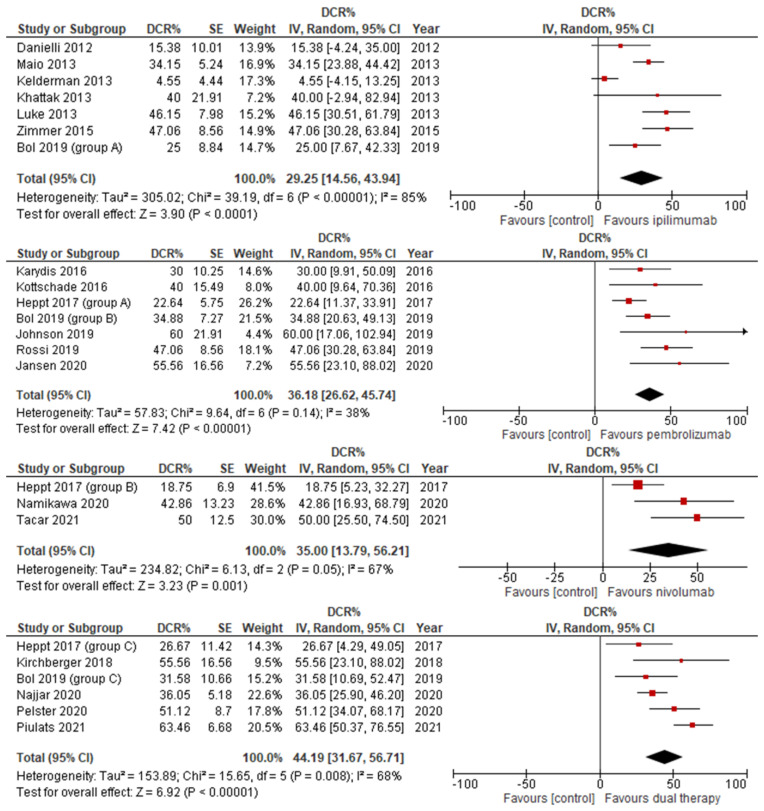
Disease control rate stratified by ICI therapy.

All therapies exhibited a statistically significant increase in DCR. DCR varied markedly less than ORR between the different ICIs. The greatest improvement was seen in dual therapy with a DCR of 44.19 (CI 31.6–56.71), followed by pembrolizumab at 36.18 (CI 26.62–45.74). Next was nivolumab with a total DCR of 35.00; notably, nivolumab had much larger confidence intervals, falling between 13.79 and 56.12, approximately double the width of the other therapies. Finally, Ipilimumab proved the most subtle improvement in DCR at 29.92 (CI 14.56–43.94). These findings are depicted in Figure 4.

#### 3.3.3. Median Progression-Free Survival

**Figure 5:**
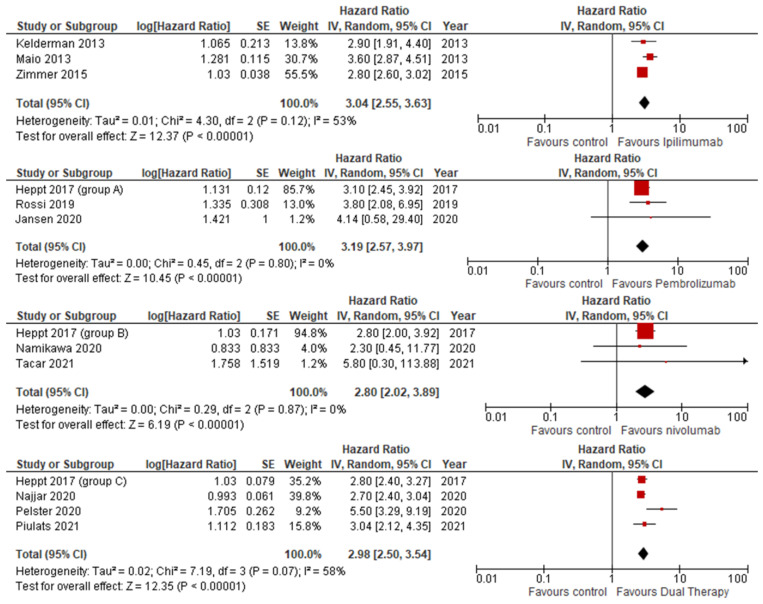
Median progression-free survival stratified by ICI therapy.

There was a relative paucity of research available to compare MPFS, with only three for single therapies and four for dual. Furthermore, for pembrolizumab and nivolumab, the majority of the weighting was contributed by Heppt et al. [28], contributing at least 85.7%. Despite this, similar results were seen across all therapies, each identifying a statistically significant improvement in MPFS. The greatest improvement was seen in pembrolizumab at 3.19, followed by Ipilimumab at 3.04, dual therapy at 2.98, and finally in nivolumab, scoring 2.80. Figure 5 illustrates these findings.

#### 3.3.4. Adverse Events

**Figure 6:**
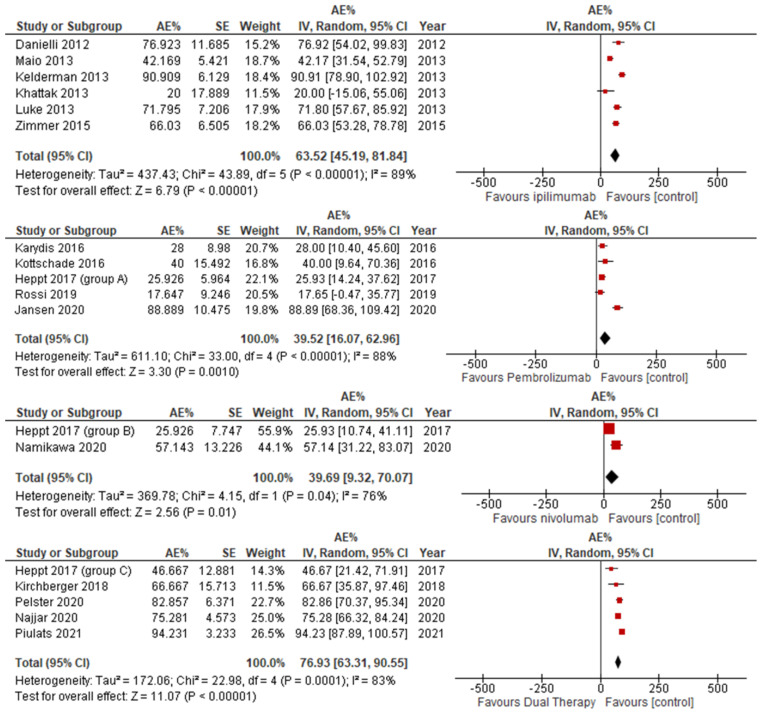
Adverse event rate stratified by ICI therapy.

Adverse events were noted across all cohorts regardless of treatment regime. Pembrolizumab was the safest ICI with an adverse event rate of 39.52, closely followed by nivolumab at 39.69; however, nivolumab had much less research conducted into adverse events, with only two applicable studies. Next was Ipilimumab at 63.52, and, finally, dual therapy had the most adverse events with a total value of 76.93, as depicted in Figure 6.

#### 3.3.5. Summary of Outcomes

Below is a summary table comparing the different ICIs across each outcome, displayed in Table 2.

## 4. Discussion

### 4.1. Analysis

Although research into the efficacy of ICI therapy in mUM remains relatively limited, the majority of existing studies demonstrate that ICI therapy is effective for treatment [10,38,39]. Existing analysis suggests that dual immune checkpoint inhibition (ICI), such as the combination of anti-CTLA-4 and anti-PD-1 therapies, is superior to single-agent ICI therapy in terms of overall efficacy. Specifically, dual ICI has demonstrated improved objective response rates (ORR) and disease control rates (DCR) across several clinical trials [10,40,41]. This meta-analysis substantiates these findings, reinforcing the efficacy of dual ICI for ORR and DCR increases. However, it also highlights critical nuances in therapeutic outcomes and adverse event profiles that warrant closer examination, particularly when stratifying single-agent therapies and comparing them directly to dual therapy.

Notably, no systematic review or meta-analysis currently published has undertaken a comprehensive comparison of single-agent ICI therapies against one another and against dual ICI therapy. This gap in the literature is addressed in the present analysis, which provides a detailed examination of therapeutic efficacy and safety across different regimens. When stratifying single-agent ICI therapies, unexpected findings emerged. For instance, MPFS was observed to be greater with Ipilimumab and pembrolizumab compared to dual therapy. These findings challenge the prevailing notion that dual ICI is categorically superior to monotherapy, and suggest that certain single-agent therapies may offer comparable or even superior outcomes for specific endpoints. Moreover, adverse events were found to be significantly more frequent with dual therapy than with any single-agent ICI, further emphasising the importance of balancing efficacy with tolerability in clinical decision-making.

A closer inspection of the ORR funnel plots provides additional insights. The lower CI for dual therapy (8.39) was found to intersect with the upper CI for pembrolizumab (10.72) and nivolumab (10.24), indicating no statistically significant differences in ORR between these therapies. Similarly, in the DCR funnel plots, the lower CI for dual therapy (31.67) crossed the upper CIs for each of the single-agent therapies evaluated. These findings suggest that, while dual therapy may appear superior in aggregate analyses, its advantages may not be statistically significant when directly compared to specific single-agent therapies. This nuanced interpretation underscores the need for further research to identify patient subgroups that might derive the most benefit from single-agent versus dual therapy.

The implications of these findings extend beyond efficacy metrics. Safety profiles are a critical consideration in determining the clinical utility of ICI therapies, particularly in the context of metastatic disease where patients often have limited tolerance for treatment-related toxicities. Dual therapy, while associated with higher ORR and DCR in aggregate, exhibited a significantly higher incidence of adverse events compared to single-agent therapies. This raises important questions about the risk-benefit ratio of dual therapy in routine clinical practice, especially when single-agent therapies such as pembrolizumab demonstrated comparable efficacy for certain outcomes with a significantly lower adverse event burden. For example, pembrolizumab outperformed dual therapy in terms of MPFS while maintaining a more favourable safety profile, positioning it as a viable alternative to dual therapy in select clinical scenarios.

The unique perspective provided by this meta-analysis permits a more refined understanding of the relative performance of different ICI regimens. Contrary to contemporary research that frequently positions dual ICI therapy as the most effective approach, these findings suggest that single-agent therapies, when isolated and stratified by regimen, can serve as appropriate competitors to dual therapy for certain clinical outcomes. This perspective is particularly relevant considering the challenges associated with dual therapy, including its higher toxicity profile and the logistical and financial burdens it imposes on healthcare systems and patients.

The broader implications of this study extend to clinical practice and future research. First, these findings highlight the need for personalised treatment approaches that consider the unique clinical and biological characteristics of individual patients. For example, patients with a higher risk of treatment-related toxicities may benefit from single-agent therapies that offer comparable efficacy with reduced adverse event rates. Second, the observed overlap in confidence intervals for ORR and DCR between single-agent and dual therapies underscores the need for more robust, head-to-head comparative trials to elucidate the relative benefits of these approaches more definitively. Lastly, this study underscores the importance of incorporating safety and quality-of-life metrics into the evaluation of ICI therapies, ensuring that treatment decisions are informed by a comprehensive understanding of their impact on patients.

This meta-analysis provides a critical re-evaluation of the relative efficacy and safety of single-agent versus dual ICI therapies in metastatic uveal melanoma. The findings challenge prevailing assumptions about the superiority of dual therapy and highlight the potential of specific single-agent therapies, such as pembrolizumab, to achieve comparable or superior outcomes for certain endpoints while minimising adverse events. These insights contribute to a growing body of evidence that supports a more nuanced and individualised approach to the management of mUM, paving the way for future research and improved clinical decision-making.

The relationship between ICI therapies and mUM characterised in this study closely mirrors that of cutaneous melanomas not only across ORR, DCR, MPFS and AER, but also potential toxicity associated adverse events being higher in combination therapies [42,43,44]. The inclusion of cohorts with different melanoma types, grades and locations permits a far larger sample size and therefore more discrete results. However, cohorts with multiple types of melanomas diminishes external validity when investigating solely patients with mUM as each type of melanoma may respond differently to ICI therapy, presenting possible confounding. Whilst the similar trends observed aid in understanding this condition and the drawing of parallels can guide further research, the correlation in these analogous outcomes should not be used to rationalise the assumption that mUM will behave similarly to cutaneous melanomas across other outcomes or interventions. Through further research into individual melanomas including mUM, clinicians will be equipped with focussed data to better tailor treatment to a patient’s case rather than using studies conducted across a large, generalised cohort.

### 4.2. Clinical Impact of Findings

The optimal approach to managing mUM remains inadequately defined, with no consensus or established rationale guiding the selection of ICI regimens. While Tebentafusp has demonstrated clear superiority over any ICI therapy for the treatment of HLA-A0201-positive mUM, the question of the most appropriate ICI therapy for HLA-A0201-negative patients persists unresolved [13].

The findings of this meta-analysis provide valuable insights into the comparative efficacy and safety of different ICI regimens. By delineating the outcomes associated with various single-agent therapies and their performances relative to dual therapy, this study offers clinicians a more nuanced understanding of the therapeutic landscape. Such insights enable healthcare providers to tailor treatment regimens more precisely to the clinical characteristics of individual patients. Furthermore, this evidence empowers clinicians to offer patients more specific and evidence-based information about the potential benefits, risks, and expected outcomes of proposed therapies, thereby facilitating shared decision-making and personalised care

### 4.3. Scope for Future Research

The treatment of mUM remains a field with a notable scarcity of research. This limitation was particularly evident for nivolumab, which had the fewest studies and the smallest number of participants across all outcomes assessed. Similarly, the outcome of median progression-free survival was associated with significantly fewer studies compared to other metrics, highlighting a critical gap in the evidence base.

Expanding research efforts in these areas is essential to enhance the accuracy and precision with which various ICI regimens are evaluated. Such advancements hold the potential to optimise clinical decision-making and improve outcomes for patients affected by this challenging condition. With sufficient research, poorly performing ICI therapies could be identified and potentially eliminated from clinical consideration. Additionally, further analysis of cost-utility would afford more insight into whether the differing costs of different ICI therapies is substantiated by these changes in outcomes. Over time, this could pave the way for the development of a gold-standard treatment framework, incorporating a stepwise approach to therapy selection. Establishing such a standardised protocol would provide clinicians with a more consistent and evidence-based strategy for managing mUM, ultimately benefiting patient care. However, achieving this level of standardisation is contingent upon further extensive and rigorous research in this domain.

### 4.4. Limitations

Due to the nature of the research conducted, there are several limitations identified with this meta-analysis. Firstly, there is significant variation in the amount of literature available for each therapy, with nivolumab having half the number of studies compared to any other treatment. The resultant range in sample sizes introduces possible skew in the results, impairing the ability to make an apt comparison between ICI regimes. The cause of the relative paucity of studies reviewing nivolumab specifically is unclear. Within the literature search nivolumab studies were disproportionately excluded due to use of multiple ICIs within the treatment cohort. Although removing this exclusion criterion may have allowed for more equivocal sample sizes between the therapies, the data extraction would not have been possible as outcomes could not be attributed to a single therapy as required. Additionally, a similar lack of nivolumab studies has been observed in other meta-analyses investigating ICIs in mUM, suggesting the cause for fewer nivolumab studies being yielded extends beyond the methodology of this study and represents a systemic scarcity of investigation [10].

Furthermore, information pertaining to adverse events is limited. In this meta-analysis, all adverse events are considered equal with even weighting in outcome assessment, where life threatening grade 4 events such as sepsis and haemorrhage are far more clinically significant than mild grade 1 events such as fatigue or transient confusion [45]. As a result, this meta-analysis provides a relatively limited perspective into the safety profile of each regime.

## 5. Conclusions

This meta-analysis highlights the nuanced efficacy and safety profiles of ICI therapies in mUM, shedding light on critical distinctions between single-agent and dual ICI regimens. While dual ICI therapy demonstrated superior ORR and DCR, single-agent therapies such as pembrolizumab showed comparable MPFS with significantly lower AER. Importantly, confidence interval analyses revealed overlapping outcomes between dual therapy and certain single-agent therapies, suggesting that dual ICI is not unequivocally superior in all clinical contexts. These findings challenge prevailing assumptions about the uniform superiority of dual therapy and emphasise the need for personalised treatment approaches that balance efficacy with tolerability. Furthermore, the scarcity of research on specific regimens, particularly nivolumab and MPFS outcomes, underscores the urgent need for more robust, head-to-head comparative trials. By refining the understanding of how ICI therapies perform in mUM, this analysis lays the groundwork for more informed clinical decision-making and the potential development of standardised, evidence-based treatment algorithms.

## Figures and Tables

**Table 1:** Results of Literature Search.

Author	Year	ICI Regime	Type of Study	Number of Participants	ORR	MPFS	DCR %	AE
Danielli [19]	2012	Ipilimumab	Prospective	13	0	N/A	15.38	10
Kelderman [20]	2013	Ipilimumab	Prospective	22	4.55	2.9	4.55	20
Khattak [21]	2013	Ipilimumab	Retrospective	5	0	N/A	40	1
Luke [22]	2013	Ipilimumab	Retrospective	39	2.56	N/A	46.15	28
Maio [23]	2013	Ipilimumab	Prospective	83	4.88	3.6	34.15	35
Zimmer [24]	2015	Ipilimumab	Phase 2 clinical trial	53	0	2.8	47.06	35
Bol et al. group A [25]	2019	Ipilimumab	Retrospective	24	0	N/A	25	N/A
Karydis [26]	2016	Pembrolizumab	Retrospective	25	5	N/A	30	7
Kottschade [27]	2016	Pembrolizumab	Prospective	10	30	N/A	40	4
Heppt Group A [28]	2017	Pembrolizumab	Retrospective	54	5.66	3.1	22.64	14
Bol Group B [25]	2019	Pembrolizumab	Retrospective	43	6.98	N/A	34.88	N/A
Johnson [29]	2019	Pembrolizumab	Phase 2 clinical trial	5	20	N/A	60	N/A
Rossi [30]	2019	Pembrolizumab	Prospective	17	11.76	3.8	47.06	3
Jansen [31]	2020	Pembrolizumab	Prospective	9	0	4.1	55.56	8
Heppt Group B [28]	2017	Nivolumab	Retrospective	32	3.13	2.8	18.75	13
Namikawa [32]	2020	Nivolumab	Retrospective	14	7.14	2.3	42.86	8
Tacar [33]	2021	Nivolumab	Retrospective	16	18.75	10	50	N/A
Heppt Group C [28]	2017	Dual	Retrospective	15	13.33	2.8	26.67	7
Kirchberger [34]	2018	Dual	Retrospective	9	0	N/A	55.56	6
Bol Group C [25]	2019	Dual	Retrospective	19	21.05	N/A	31.58	N/A
Najjar [35]	2020	Dual	Retrospective	89	11.63	2.7	36.05	67
Pelster [36]	2020	Dual	Phase 2 clinical trial	35	18.18	5.5	51.52	29
Piulats [37]	2021	Dual	Phase 2 clinical trial	52	11.54	3	63.46	49

**Table 2:** Summary comparison table.

	1st	2nd	3rd	4th
ORR	Dual therapy	Pembrolizumab	Nivolumab	Ipilimumab
DCR	Dual therapy	Pembrolizumab	Nivolumab	Ipilimumab
MPFS	Pembrolizumab	Ipilimumab	Nivolumab	Dual therapy
AER	Pembrolizumab	Nivolumab	Ipilimumab	Dual therapy

## Data Availability

Additional data can be found in Appendix A.

## Abbreviations

The following abbreviations are used in this manuscript:

mUM	Metastatic uveal melanoma
ICI	Immune checkpoint inhibitor
ORR	Objective response rate
DCR	Disease control rate
MPFS	Median progression-free survival
AER	Adverse event rate
CTLA-4	Cytotoxic T-lymphocyte-associated antigen 4
PD-1	Programmed cell death protein 1

## Appendix A

**Table A1:** Search items used in the literature search.

Group 1	Group 2	Group 3
Metastatic uveal melanoma	Immune checkpoint inhibitor	Objective response
mUM	ICI	Disease control
Uveal melanoma	Ipilimumab	Median Progression-free survival
Choroidal melanoma	Pembrolizumab	Adverse event
Ocular melanoma	Nivolumab	Assessment, patient outcomes
Eye neoplasms	Dual therapy	Drug-related side effects
	Programmed death protein 1, human	
	CTLA 4 Ig	
	Induction chemotherapy	
